# ESA-YOLO: An efficient scale-aware traffic sign detection algorithm based on YOLOv11 under adverse weather conditions

**DOI:** 10.1371/journal.pone.0336863

**Published:** 2025-11-14

**Authors:** ChenHao Li, ShuXian Liu, ZiNuo Peng

**Affiliations:** School of Computer Science and Technology, XinJiang University, Urumqi City, Xinjiang Autonomous Region, China; Hohai University, CHINA

## Abstract

Traffic sign detection is a critical component of autonomous driving and advanced driver assistance systems, yet challenges persist in achieving high accuracy while maintaining efficiency, particularly for multi-scale and small objects in complex scenes. This paper proposes an improved YOLOv11-based traffic sign detection algorithm that tackles above challenges through three key innovations: (1) A Dense Multi-path Feature Pyramid Network (DMFPN) that boosts multi-scale feature fusion by enabling comprehensive bidirectional interaction between high-level semantic and low-level spatial information, augmented by a dynamic weighted fusion mechanism. (2) A Context-Aware Gating Block (CAGB) that efficiently integrates local and global contextual information through lightweight token and channel mixer, enhancing the small-object detection ability without excessive computational overhead. (3) An Adaptive Scene Perception Head (ASPH) that synergistically combines multi-scale feature extraction with attention mechanisms to improve robustness in adverse weather condition. Extensive experiments on the TT100K and CCTSDB2021 datasets demonstrate the model’s superior performance. On the TT100K dataset, our model outperforms the state-of-the-art YOLOv11n model, achieving improvements of 3.8% in mAP@50 and 3.9% in mAP@50-95 while maintaining comparable computational complexity and reducing parameters by 20%. Similar gains are observed on the CCTSDB2021 dataset, with enhancements of 2.3% in mAP@50 and 1.8% in mAP@50-95. Furthermore, experimental results also demonstrate that our proposed model exhibits superior performance in small object detection and robustness in complex environments compared to mainstream competitors.

## Introduction

The traffic sign detection system is a crucial component of both autonomous driving systems and advanced driver-assistance systems. A key challenge in the practical deployment of intelligent driving systems lies in how to further enhance the accuracy of traffic sign detection while ensuring real-time performance.

Algorithms for object detection based on deep learning can be broadly categorized into two classes: single-stage and two-stage approaches. Two-stage detectors, represented by Faster R-CNN [1], exhibit superior detection accuracy. However, their inference speed is inherently limited, making them unsuitable for real-time traffic sign detection systems. Single-stage detectors primarily consist of two branches, one is the YOLO series of algorithms and the other is transformer-based detectors, exemplified by DETR [2] and its subsequent variants.

Thanks to the long-range dependencies enabled by attention mechanisms, Transformer-based object detectors exhibit exceptionally powerful feature extraction capabilities. However, in their early stages, much like two-stage detectors, they suffered from slow inference speeds. It was not until the introduction of TR-DETR [3] that real-time performance was achieved for the first time. Moreover, such models typically require massive parameter counts and computational overhead, making them poorly suited for deployment on in-vehicle mobile devices with limited resources.

The YOLO series algorithms, renowned for their optimal speed-accuracy trade-off, are highly suitable for real-world deployment in object detection tasks. However, it is suboptimal to directly apply YOLO algorithms to traffic sign detection systems, as they lack domain-specific optimizations for the unique challenges in this field.

First, since in-vehicle cameras perform dynamic real-time capture, the distance between the vehicle and traffic signs varies continuously, resulting in significant scale variation of target objects within the captured images. This imposes higher demands on the multi-scale detection capability of the model. However, the PANet-based feature pyramid commonly adopted in YOLO series models relies on unidirectional and oversimplified fusion paths, which inevitably leads to partial detail loss during feature aggregation. Moreover, the model lacks sufficient utilization of shallow feature layers, thereby weakening its small object detection ability-a critical capability for traffic sign detection systems that frequently encounter numerous tiny target objects. Additionally, complex environment is another major challenge in traffic sign detection. Maintaining the model’s focus on target objects amid cluttered scenes remains a key issue to be addressed.

This paper proposes a traffic sign detection algorithm based on an improved YOLOv11, aiming to enhance multi-scale and small object detection performance, as well as robustness in complex scenarios, while maintaining competitive computational efficiency and parameter size.

The main contributions of our work are summarized as follows:

1) Dense Multi-path Feature Pyramid Network (DMFPN), a novel architecture that enables comprehensive bidirectional fusion of high-level and low-level features. This design minimizes information loss during feature map generation and maximizes utilization of shallow-layer features while maintaining computational efficiency, thereby significantly enhancing multi-scale and small object detection performance.

2) Context-Aware Gating Block (CAGB), a dedicated feature extraction module, which is specifically designed to enhance small-scale object detection capability by capturing and integrating richer contextual information.

3) A multi-scale attention head, named Adaptive Scene Perception Head (ASPH), is proposed in this paper that synergistically combines multi-scale feature extraction with attention mechanisms to enhance model adaptability in complex scenes.

## Related work

### Traffic Sign detectors

In the early stages of research, traffic sign detection algorithms were predominantly based on traditional machine learning methods. In 2015, Hechri *et al*. [4] presented a highly robust road lane and traffic sign recognition method that combines image processing with pattern recognition techniques. In 2017, Huang Z *et al*. [5] developed an efficient traffic sign recognition approach based on the Extreme Learning Machine (ELM). By leveraging the fast training and effective classification capabilities of ELM, and optimizing the network structure and parameters, this method achieved significant improvements in both recognition speed and accuracy. However, in recent years, the rapid advancement of deep learning has demonstrated superior performance in the field of computer vision compared to traditional machine learning methods, while significantly reducing the complexity of algorithm.

Deep learning-based object detection algorithms can generally be categorized into two types: single-stage and two-stage detectors. Single-stage object detectors, represented by the YOLO series, reformulate object detection as a regression task, eliminating the necessity for region proposal networks to generate anchor boxes. This paradigm shift significantly accelerates the model inference speed while maintaining competitive detection accuracy. Therefore, at the current stage, a substantial portion of research on traffic sign detection algorithms is conducted based on the YOLO series. In this section, we will systematically review the most critical and mainstream versions of the YOLO algorithms, as well as various traffic sign detectors developed upon them.

In 2016, Redmon *et al*. [6] introduced the YOLOv1, the first work in the YOLO series, which became the first real-time, end-to-end object detector. In 2020, YOLOv5, introduced by Glenn Jocher *et al*., implemented adaptive image scaling and pioneered scalable model architectures. Shenming Qu *et al*. [7] proposed a traffic sign detection algorithm based on YOLOv5 for complex weather conditions. They incorporated a coordinate attention mechanism to enhance the network’s feature extraction capability, enabling the capture of more dense spatial information. Additionally, an extra detection head was added in the shallow layers to improve the network’s ability to detect small-scale targets. Junfan Wang *et al*. [8] recognized the multi-scale challenges inherent in traffic sign detection and proposed improvements based on YOLOv5. They introduced the AF-FPN architecture, which comprises two key components: an Adaptive Attention Module (AAM) and a Feature Enhancement Module (FEM), designed to address scale variation and enhance feature representation.Also based on YOLOv5, Liwei Liu *et al*. [8] proposed a lightweight traffic sign detection algorithm that significantly reduces the model’s parameter count and computational complexity.

In 2023, YOLOv8 [9], developed by Ultralytics company, introduced four major innovations: an enhanced backbone network, a decoupled head structure, Distribution Focal Loss (DFL) and an anchor-free detection paradigm. Guobo Xie *et al*. [10] proposed GRFS-YOLO, an efficient multi-scale traffic sign detection algorithm based on YOLOv8, which achieves a more extreme level of model lightweighting. However, this comes at the cost of a relatively noticeable decrease in accuracy. Qian Shen *et al*. [11] proposed CSW-YOLO, a small-object traffic sign detection algorithm based on YOLOv8. Their approach primarily introduces the Large Separable Kernel Attention (LSKA) [12] mechanism to expand the receptive field, thereby capturing richer contextual information to enhance small-object detection. Additionally, an extra detection head dedicated to small objects was incorporated. To offset the increased parameters and computational overhead resulting from these improvements, the authors integrated the Faster-Block module from FasterNet [13], replacing the Bottleneck structure within the C2f module to achieve a balanced trade-off.

### Multi-scale features fusion for object detection

Feature Pyramid Networks (FPN) [14] are an effective approach for addressing the challenge of large scale variations of targets in traffic sign detection tasks. FPN is a widely adopted architecture in neural networks designed for multi-scale feature extraction. Its core idea lies in constructing feature layers at different resolutions and fusing high-level semantic information with low-level detailed information, thereby enhancing the model’s ability to handle objects of varying scales.

The concept of FPN was first introduced by He *et al*. in 2017. This approach fused semantic information from high-level feature maps with spatial details from low-level feature maps, constructing feature representations with rich multi-scale discriminability, thereby significantly enhancing model performance in multi-scale object detection tasks. In 2018, Liu *et al*. proposed Path Aggregation Network (PANet) [15], which enhanced the original FPN architecture by introducing an additional bottom-up pathway. In 2020, Liu *et al*. introduced the Bidirectional Feature Pyramid Network (BiFPN) [16], which enhanced multi-scale object detection through learnable cross-scale feature fusion and bidirectional information flow. Qiao *et al*. proposed Recursive-FPN [17], which enhanced cross-scale feature interaction through recursive stacking of multi-level feature fusion modules.

In 2021, Jiang *et al*. introduced Giraffe feature pyramid network (GFPN) [18] that promoted rich information sharing across various spatial scales and simultaneously among different levels of latent semantics. In 2023, Yang *et al*. proposed an asymptotic feature pyramid network (AFPN) [19] to support direct interaction at non-adjacent levels to avoid the loss or degradation of feature information. Xu *et al*. proposed Efficient RepGFPN [20] by optimizing the multi-scale feature fusion strategy and introducing a dynamic channel allocation mechanism in GFPN. In 2024, Zhang *et al*. proposed a versatile neck named Multi-Branch Auxiliary FPN (MAFPN) [21] to efficiently integrate multi-scale features. Chen *et al*. proposed High-level Screening-feature Fusion Pyramid (HS-FPN) [22], which facilitated multi-level feature fusion by employing high-level semantic features as adaptive weights to refine low-level features through channel attention.

### Attention mechanism

Attention mechanisms are vital for enhancing small object detection and strengthening model robustness, which are key to effective traffic sign detection. Originally developed for neural machine translation, attention mechanisms have been widely adopted in computer vision due to their seamless integration into CNN backbone architectures. These mechanisms capture long-range dependencies and generate attention feature maps that emphasize task-relevant regions.

Squeeze-and-Excitation Networks (SENet) [23] introduced channel-wise attention mechanisms for the first time. Efficient Channel Attention (ECA) [24] replaced the fully connected layers following global average pooling with 1×1 convolutions, eliminating dimensionality reduction while more efficiently capturing cross-channel interactions. Convolutional Block Attention Module (CBAM) [25] pioneered the sequential integration of channel and spatial attention mechanisms, enabling simultaneous attention allocation across both dimensions. Global Context Network (GCNet) [26] simplified non-local self-attention by computing a single shared global context for all positions. Coordinate Attention (CA) [27] decomposed channel attention into horizontal and vertical positional-aware components, preserving location sensitivity while capturing channel-wise interdependencies.Stand-Alone Self-Attention [28] demonstrated the potential in vision tasks by replacing traditional convolutional layers exclusively with self-attention layers. The Separable Self-attention (SSA) [29] mechanism decomposed the conventional global self-attention in Vision Transformers (ViTs) into two components: Local Group Self-attention (LG-SA) and lightweight Global Token Propagation (GTP). This approach substantially reduced computational complexity while preserving the capacity for global context modeling. Swift Attention (SA) [30] employd learnable additive interactions between tokens, eliminating the need for explicit pairwise similarity calculations.

## The proposed method

### ESA-YOLO traffic sign detection algorithm

**Fig 1:**
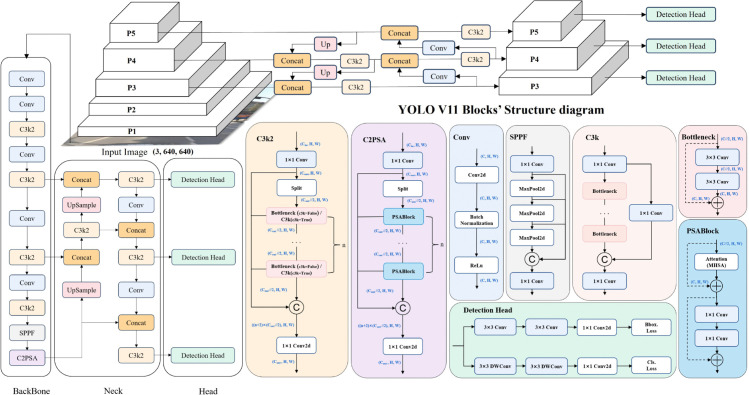
The network structure diagram of YOLOv11.

The YOLOv11 model, developed by Ultralytics company as an enhanced version of YOLOv8, exhibits superior accuracy with reduced parameters and computational costs, establishing itself as a new state-of-the-art model. Consequently, we selected YOLOv11 as our baseline framework. The architecture of YOLOv11 is illustrated in Fig 1.

**Fig 2:**
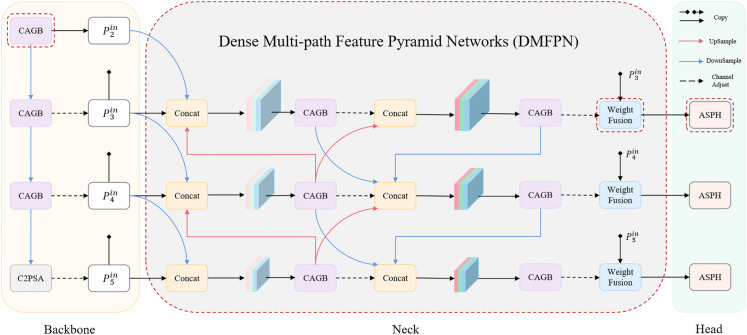
The network structure diagram of ESA-YOLO.

To enhance multi-scale detection performance and small object recognition accuracy while improving model robustness in complex backgrounds, we propose several key improvements to YOLOv11 and introduce a novel traffic sign detection algorithm named Efficient Scale-Aware YOLO (ESA-YOLO). ESA-YOLO achieves an optimal balance between computational efficiency and detection accuracy.The ESA-YOLO network architecture is illustrated in Fig 2.

Firstly, to overcome the shortcoming that the original neck network fails to fully leverage the multi-scale feature information and lacks effective integration of shallow-level features, we replace the PANet structure with the dense multi-path Feature Pyramid Network (DMFPN) proposed in this paper to improve the detection performance for multi-scale and small traffic signs. Secondly, it can be observed that the C3K2 module is ubiquitously employed in neural networks for feature extraction, constituting the primary source of both computational overhead and model parameters. To enhance the capability of extracting features with minimal computational overhead and parameters, we propose a context-aware gating block (CAGB) as a computationally efficient alternative to the conventional C3K2 module. Thirdly, we propose an adaptive scene perception head (ASPH) to model multi-scale features and global dependencies, improving robustness in complex scenes.

### Dense multi-path feature pyramid network

The Path Aggregation Feature Pyramid Network (PAFPN), despites its extensive integration into YOLO architectures as a feature fusion mechanism, still possesses three notable limitations which constrain the network’s capacity for further enhancement in multi-scale object detection performance.

**Fig 3:**
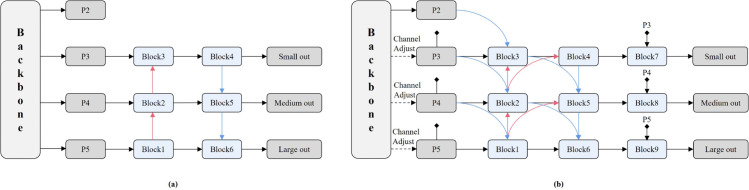
PANet and DMFPN.

Firstly, the limitations of PANet stem from its inherently unidirectional and oversimplified fusion pathways, which fail to fully leverage the multi-scale feature information generated across preceding stages during feature fusion. As illustrated in Fig 3(a), taking Block5 as an example, it only fuses feature from the sibling 4P layer and the downsampled upper node, while failing to incorporate stage-wise multi-scale information from Block1 and Block3, which inherently encapsulate precise localization cues and rich semantic representations, respectively. Similarly, Block4 and Block6 exhibit the same issue, resulting in suboptimal feature integration and reduced representation capacity. Secondly, PANet lacks effective integration of shallow-level features, which inherently encode fine-grained spatial details and high-frequency visual patterns critical for small object detection and precise boundary localization. For instance, in the Block2, the input exclusively incorporates the up-sampled P5 layer and its sibling P4 layer, while neglecting the critical contribution of shallow low-level spatial information contained within the P3 layer. Thirdly, PANet employs a strategy that progressively increases the number of channels during downsampling while decreasing them during upsampling. However, this approach may introduce feature redundancy that compromises feature fusion effectiveness, and exhibits inherent incompatibility with the requirements of our detection task.

To address the aforementioned limitations, this paper proposes Dense Multi-path Feature Pyramid Network (DMFPN), a novel neck architecture that enables comprehensive fusion of both shallow and deep feature representations while maintaining computational efficiency.

PANet adopts a pyramidal channel allocation strategy, where deeper layers are assigned more channels. This design stems from the assumption that high-level features require more channels to encode semantic information, while reducing the channel numbers of larger low-level feature maps can effectively decrease computational costs. However, feature maps of different scales often exhibit redundant information in their channels. Blindly increasing channel numbers may lead to high-dimensional channels learning redundant or noisy features. Furthermore, when deep layers contain excessive channels, the dominance of high-level semantic features may overwhelm low-level detailed features, causing the model to overly focus on the semantic information of large objects while neglecting the fine details of small objects—this contradicts the objective of our detection task. Therefore, as illustrated in Fig 3(b), unlike PANet, we first apply a uniform channel adjustment to all input feature maps of varying scales before feeding them into the neck network, while ensuring their consistency after each subsequent feature fusion. This approach offers several advantages. Firstly, uniform channel numbers balance the contributions of features across different scales, preventing information loss or misalignment due to channel inconsistency, thereby improving feature fusion efficiency. Secondly, adjusting feature map channels effectively balances the additional computational burden introduced by subsequent dense multi-path fusion in the neck network. Thirdly, it facilitates the final weighted fusion operation by avoiding extra dimensionality expansion or reduction during feature fusion, thereby reducing computational complexity and potential information loss.

Shallow features inherently preserve rich spatial details, which are particularly critical for small object detection. To leverage this property, we prioritize the integration of shallow backbone features during the initial fusion stage. As illustrated in Fig 3(b), we enhance the originally single-input Block1 by integrating an additional input from the P4 layer of the backbone network. Similarly, Block2 and Block3 are augmented with inputs from the P3 and P2 backbone layers, respectively. The feature maps generated during the first fusion stage in the neck network can be formulated as:

$$P_{5}^{N1}=Conv(Concat(resize(P_{4}^{in}),P_{5}^{in}))$$ (1)

$$P_{4}^{N1}=Conv(Concat(resize(P_{3}^{in}),P_{4}^{in},resize(P_{5}^{N1})))$$ (2)

$$P_{3}^{N1}=Conv(Concat(resize(P_{2}^{in}),P_{3}^{in},resize(P_{4}^{N1})))$$ (3)

The input from backbone can be represented as $P_{i}^{\text{in}}$, where i∈3,4,5 indexes the hierarchical position in the feature pyramid. The feature maps generated within the neck network can be represented as $P_{i}^{Nj}$, where j∈1,2 indexes the fusion stage and i∈3,4,5 indexes the hierarchical position in the feature pyramid. Conv() represents the convolutional operation, which performs either channel dimension adjustment or feature extraction. Concat() represents the channel-wise concatenation operation, which merges feature maps along the channel dimension. resize() represents the upsampling or downsampling operation, which aligns the spatial dimensions of feature maps.

To effectively handle detection tasks involving pronounced multi-scale object characteristics, the second-stage feature fusion in DMFPN performs more intensive multi-branch fusion compared to PANet. This design fully exploits multi-resolution feature maps generated during fusion to enhance cross-layer feature interaction. As illustrated in Fig 3(b), Block5 integrates four-branch inputs from Block1, Block2, Block3, and Block4, significantly enriching the source diversity of the fused feature flow. Furthermore, Block4 absorbs higher-level semantic features from Block2, while Block6 refines spatial localization accuracy with complementary Block2 features. The feature maps generated during the second fusion stage in the neck network can be formulated as:

$$P_{3}^{N2}=Conv(Concat(P_{3}^{N1},resize(P_{4}^{N1})))$$ (4)

$$P_{4}^{N2}=Conv(Concat(resize(P_{3}^{N1}),P_{4}^{N1},resize(P_{2}^{N1}),resize(P_{3}^{N2})))$$ (5)

$$P_{5}^{N2}=Conv(Concat(resize(P_{4}^{N1}),P_{5}^{N1},resize(P_{4}^{N2})))$$ (6)

The feature maps generated within the neck network can be represented as $P_{i}^{Nj}$, where j∈1,2 and i∈3,4,5. Conv() represents the convolutional operation, which performs either channel dimension adjustment or feature extraction. Concat() represents the channel-wise concatenation operation, which merges feature maps along the channel dimension. resize() represents the upsampling or downsampling operation, which aligns the spatial dimensions of feature maps.

The contribution of different feature maps to fusion varies significantly. Simply concatenating features may yield suboptimal fusion results. To adaptively learn the importance of each feature, DMFPN introduces a weighted dynamic feature fusion mechanism. As illustrated in Fig 3(b), we first adjust the channel dimensions of input feature maps from different backbone layers to control model parameters and computational costs. These features are then fused with their corresponding second-stage neck outputs via learned weights to produce the final output feature maps. This process not only enables discriminative fusion of input feature maps but also functions analogously to identity mapping in neural networks, preserving critical gradient flow and feature representational capacity. There exist three prevalent weighted fusion strategies.

The first is Unbounded Fusion, which employs a straightforward learnable weight parameter. However, due to its unconstrained nature, this approach may lead to training instability. Unbounded Fusion can be formulated as:

$$𝐨=\sum_{i}^{\;}w_{i}*I_{i}$$ (7)

*w* indexes the learnable weight parameter, *I* indexes the feature map.

The second is Softmax-based Fusion, which confines weights to the range [0, 1] to ensure training stability, albeit at the cost of slower convergence. Softmax-based Fusion can be formulated as:

$$𝐨=\sum_{i}^{\;}\frac{e^{w_{i}}*I_{i}}{\epsilon +\sum_{j}^{\;}e^{w_{i}}}$$ (8)

*w* indexes the learnable weight parameter, *I* indexes the feature map. $\epsilon \in R^{+}$ be a vanishingly small constant (0<ϵ≪1). e be a constant (2 < *e* < 3).

The third, Fast Normalized Fusion, achieves both bounded outputs [0, 1] and rapid training efficiency through a Softmax-like normalization. Given these advantages, our framework adopts the third approach for weighted feature fusion. Fast Normalized Fusion can be formulated as:

$$𝐨=\sum_{i}^{\;}\frac{w_{i}*I_{i}}{\epsilon +\sum_{j}^{\;}w_{j}}$$ (9)

*w* indexes the learnable weight parameter, *I* indexes the feature map. $\epsilon \in R^{+}$ be a vanishingly small constant (0<ϵ≪1).

The final output of the neck network can be formulated as:

$$P_{3}^{out}=\frac{w_{1}*P_{3}^{in}+w_{2}*P_{3}^{N2}}{w_{1}+w_{2}+\epsilon }$$ (10)

$$P_{4}^{out}=\frac{w_{1}^{{\;}^{\prime }}*P_{4}^{in}+w_{2}^{{\;}^{\prime }}*P_{4}^{N2}}{w_{1}^{{\;}^{\prime }}+w_{2}^{{\;}^{\prime }}+\epsilon }$$ (11)

$$P_{4}^{out}=\frac{w_{1}^{{\;}^{\prime \prime }}*P_{5}^{in}+w_{2}^{{\;}^{\prime \prime }}*P_{5}^{N2}}{w_{1}^{{\;}^{\prime \prime }}+w_{2}^{{\;}^{\prime \prime }}+\epsilon }$$ (12)

The final output of the neck can be represented as $P_{i}^{out}$, where i∈3,4,5 indexes the hierarchical position in the feature pyramid. The feature maps generated within the neck network can be represented as $P_{i}^{Nj}$, where j∈1,2 and i∈3,4,5. $w_{i},w_{i}^{{\;}^{\prime }},w_{i}^{{\;}^{\prime \prime }}$ indexes the learnable weight parameter, where i∈1,2.

### Context-aware gating block

Local and global contexts play distinct yet critical roles in object detection. Local context encodes high-frequency information from neighboring pixels, where fine-grained details are essential for precise boundary localization—particularly vital when handling occluded or small-scale objects. However, reliance on local features alone remains insufficient for robust perception. Global contextual information encodes critical scene-level semantics, enabling the model to infer object relationships, resolve ambiguities in localized feature representations, and suppress inconsistent detections through holistic scene understanding—capabilities that are particularly vital in complex environments.

Consequently, the design of feature extraction modules must explicitly account for capturing these two distinct contextual representations and their synergistic integration. Local context can typically be extracted via convolutional kernels of varying receptive fields (e.g., 3×3 or 5×5 kernels), whereas global context relies on long-range dependency modeling enabled by self-attention mechanisms [31].

**Fig 4:**
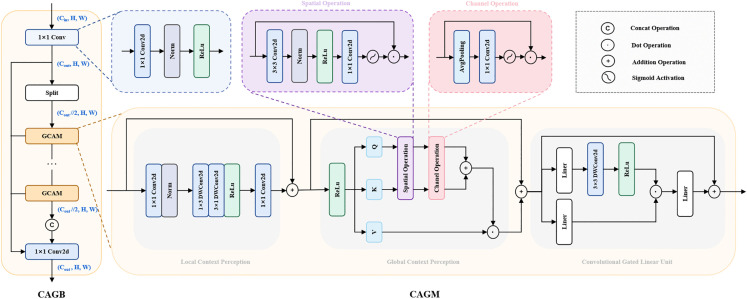
The details of CAGB and CAGM.

Motivated by these principles, we propose the Context-Aware Gating Block (CAGB) with dedicated architectural innovations, which is illustrated in Fig 4. The CAGB architecture shares similarities with C2f. The input first passes through an initial convolutional layer, followed by a split into two branches. One branch directly routes features to the output, while the other processes feature through multiple Context-Aware Gating Modules (CAGM). The two branches are then concatenated along the channel dimension and fused via a final convolutional layer to produce the output.

The CAGB module is primarily composed of CAGM, which integrates three key components: local context perception, global context perception and convolutional gated linear unit. The Local Context Perception stage extracts fine-grained local context from target-adjacent regions, while the Global Context Perception stage employs advanced token mixers to model global contextual dependencies. Finally, the Convolutional Gated Linear Unit stage performs non-linear feature transformation and dimensional adaptation to achieve local and global context aggregation.

Within the local context perception stage, the input features first undergo channel-wise transformation via a 1×1 convolutional layer. Subsequently, the features pass through ReLU-activated 1×3 and 3×1 depthwise convolutional layers, followed by another 1×1 convolution for channel dimension adjustment. This design effectively expands the receptive field to capture local contextual information, with minimal computational overhead.

The scaled dot-product attention mechanism is an effective mean of capturing long-range dependencies. However, its huge computational cost can impose an excessive burden on convolutional-based neural networks.Inspired by [28–31], it can be observed that the generality of self-attention and its variants lie in the necessity of interaction between spatial and channel domains. This suggests that a token mixer’s capacity to capture global contextual information depends on diverse interaction paradigms. Building upon this theoretical foundation, we integrates fundamental convolution-based spatial and channel attention mechanisms with the additive-attention mechanism [32] to develop an efficient token mixer for capturing long-range dependencies. The additive-attention mechanism can be formulated as:

$$e_{i}={𝐯}^{⊤}\tanh\left({𝐖}_{q}𝐪+{𝐖}_{k}𝐤\right).$$ (13)

We replace the linear projections in additive attention mechanisms with cascaded basic spatial and channel operations. This design enhances the model’s capability to extract critical spatial and channel features while mitigating the computational overhead caused by intensive matrix multiplications.The spatial and channel operation can be represented as:

$$Operation_{s}(x)=\sigma (Conv_{1\times 1}(Conv_{3\times 3},Norm,ReLU(x)))⊙x.$$ (14)

$$Operation_{c}(x)=\sigma (Conv_{1\times 1}(Pooling_{1\times 1}(x)))⊙x.$$ (15)

After sequentially undergoing the spatial and channel operation, the output feature map from the entire Global Context Perception stage can be formulated as:

$$\Phi (x)=Operation_{s}(x)+Operation_{c}(x)$$ (16)

$$𝐎(x)=\tanh(Conv_{3\times 3}(\Phi (W_{q}x)+\Phi (W_{k}x)))⊙W_{v}x$$ (17)

Where *x* denotes the input feature map and *W* represents the weight matrix. *Φ*(⋅) denotes the combination of spatial and channel operation. tanh(⋅) $\in R^{N\times C}$ represents the linear transformation applied to aggregate contextual information.

**Fig 5:**
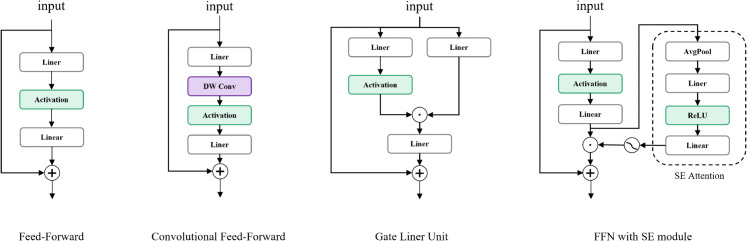
Previous channel mixer designs.

In the final stage, an advanced channel mixer is required to integrate the contextual information obtained from the previous two stages. Therefore ,we review the previous channel mixers, as illustrated in Fig 5. The stacked structure of Linear-Activation-Linear layers constitutes the fundamental building block of traditional feed-forward networks. The Convolutional Feed-Forward augments the orignal FFN by incorporating a 3×3 depthwise separable convolution, effectively addressing the limited receptive field inherent in standard feed-forward architectures. The Gated Linear Unit (GLU) bifurcates the input stream: one branch processes raw data while the other applies sigmoid activation to generate gating signals. This dynamic gating mechanism enhances feature selection capability and mitigates gradient-related issues through controlled information flow. The FFN with SE module pioneers the integration of attention mechanisms into feed-forward networks, enabling global receptive field acquisition through channel-wise feature recalibration. However, this method employs a single gating signal for all feature map tokens, leading to rigid and insufficiently fine-grained channel attention.

To overcome the shortcoming of previous channel mixers, this paper introduces the Convolutional Gated Linear Unit (CGLU) [33], as the channel mixer in the final stage. CGLU enhances the standard GLU by introducing a 3×3 depthwise convolution before the gating branch to capture neighborhood features for gating signal generation. This architectural modification expands the effective receptive field, while maintaining the channel-specific flexibility of attention mechanisms. CGLU can be represented as:

$$𝐎(x)=f(Conv_{3\times 3},ReLU(f(x))⊙f(x))⊕x$$ (18)

Where *f*(⋅) represents the fully connected layer.

CAGM achieves highly efficient extraction and integration of both local and global contextual information through a three-stage coordinated workflow, significantly enhancing the network’s capability in small object detection and complex scene understanding.

### Adaptive scene perception head

In real-world driving environments, traffic sign detection systems face substantial challenges stemming from diverse adverse conditions. Illumination variations, including low-light scenarios at night, backlighting, and rain-induced specular reflections, significantly degrade image contrast, while atmospheric interference such as haze and fog reduces visibility, often causing partial loss of texture and structural features in traffic signs. These challenges are further compounded by geometric distortions (e.g., tilted signage), partial occlusions from environmental obstructions (snow, foliage, or debris), and dynamic noise interference from precipitation effects like raindrops or snowflakes.

To overcome these limitations, we propose an Adaptive Scene Perception head (ASPH) that integrates an efficient multi-scale attention (EMA) [34] into the output stage of detector, which achieves an integration of multi-scale feature extraction and attention mechanisms. The attention mechanism empowers the model to focus on target regions while mitigating distractions from nighttime glare or rainy reflections, and adaptively suppresses less informative feature channels (e.g., attenuating rain/snow noise channels). Concurrently, the multi-scale feature extraction branch provides complementary contextual information that facilitates the reconstruction of occluded target segments while simultaneously boosting detection performance for small-scale objects.

**Fig 6:**
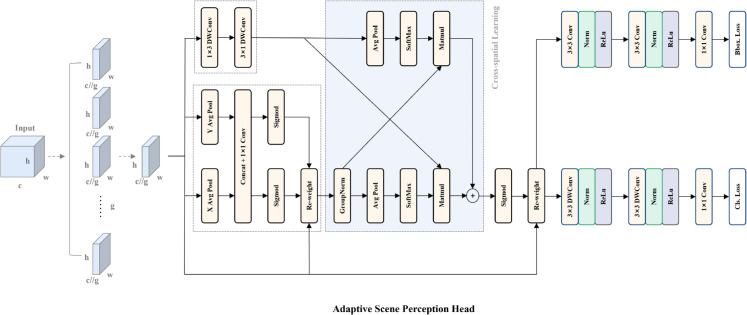
The design of ASPH.

The architecture of ASPH is illustrated in Fig 6. Given an input feature map $x\in R^{C\times H\times W}$, where *C* denotes the number of input channels, *H* and *W* represent the height and width dimensions of the input features, respectively. Firstly, *x* is partitioned into multiple groups along the channel dimension to better capture localized channel-wise distinctive features while maintaining computational efficiency and preserving fine-grained details. The grouped feature map $x^{{\;}^{\prime }}\in R^{C//G\times H\times W}$ is then processed through two parallel branches: a global feature extraction branch composed of attention mechanisms, and a local feature extraction branch employing 1×3 and 3×1 depthwise separable convolutions.

In the global feature extraction branch, the input tensor first undergoes two parallel pooling-convolution pathways: horizontal and vertical. Specifically, the horizontal pooling-convolution layer performs global average pooling along the horizontal dimension to aggregate spatial information across the vertical dimension.The value at height position *h* in the 1D feature map obtained through horizontal global average pooling can be expressed as:

$$y_{c}^{horizontal}(h)=\frac{1}{W}\sum_{0\leq i\leq W}^{\;}x_{c}(h,i)$$ (19)

Where *x*_*c*_ represents the feature map corresponding to the c-th channel. h denotes the height of the feature map. Similarly, the value at width position *w* in the 1D feature map obtained through vertical global average pooling can be expressed as:

$$y_{c}^{vertical}(w)=\frac{1}{H}\sum_{0\leq j\leq H}^{\;}x_{c}(j,w)$$ (20)

Where *x*_*c*_ represents the feature map corresponding to the c-th channel. *w* denotes the width of the feature map. These two 1D global average pooling operations capture long-range dependencies along orthogonal spatial axes while preserving more precise positional information. The resulting feature maps - each encoding distinct directional context - are concatenated and processed through a 1 × 1 convolutional layer for channel-wise feature aggregation, functionally analogous to channel attention mechanisms. The output is then split and separately transformed via sigmoid activation to produce learned attention weights along respective spatial dimensions, which are finally applied to the original input features through multiplicative gating to generate the refined output.

For the local feature extraction branch, we replaces the 3×3 standard convolution in EMA with with two consecutive depthwise separable convolutions (1 × 3 and 3 × 1). This adjustment allows network to expand the local receptive field while further reducing computational overhead, thereby effectively capturing multi-scale spatial information.

To explore interdependencies between global and local feature descriptors across all channels within each group for enhanced feature aggregation, cross-spatial learning is then conducted between the outputs of the global and local feature extraction branches. Specifically, for the output tensors from both parallel local and global branches, 2D global average pooling is employed to encode spatial context, followed by softmax normalization to generate spatial attention weight matrices. The attention weight matrices of the two branches can be formulated as:

$$W_{g}=softmax(Norm,Pooling(x_{g}))$$ (21)

$$W_{l}=softmax(Pooling(x_{l}))$$ (22)

Where *x*_*g*_ and *x*_*l*_ denote the output feature of the global and local branches, respectively, while *W*_*g*_ and *W*_*l*_ represent the corresponding attention weight matrices.

These complementary attention weight matrices are then cross-applied to their Parallel branches through matrix multiplication. The results are added then processed with a sigmoid activation to adaptively aggregate their spatial attention weight values. This process establishes pixel-wise pairwise relationships that effectively highlight global contextual dependencies across all spatial positions. The final output can be formulated as:

$$y=x⊙\sigma (\Phi (x_{g})⊗x_{l}+\Phi (x_{l})⊗x_{g})$$ (23)

Let *x* denote the original input feature map, where *x*_*g*_ and *x*_*l*_ represent the outputs of the global and local branches, respectively. The symbol *Φ* denotes the combined operation of pooling and softmax and The *σ* denotes the sigmoid activation function, while ⊗ indicates matrix multiplication.

The final output is separately fed into decoupled localization and classification branches. For precise spatial modeling, the localization branch employs stacked standard 3×3 and 1×1 convolutions to effectively capture fine-grained spatial details. The classification branch, which is inherently less sensitive to exact spatial alignment, utilizes a more efficient architecture consisting of 3×3 depthwise separable convolution followed by 1×1 standard convolution, significantly reducing computational overhead while maintaining performance. The proposed Adaptive Scene Perception Head significantly enhances small object detection accuracy and scene robustness while maintaining computational efficiency, achieved through multi-scale feature interaction and adaptive weight allocation.

## Experiment and discussion

In this section, we evaluated the performance of the ESA-YOLO on two public traffic sign detection datasets, TT100K and CCTSDB. By comparing it with other mainstream advanced detectors, we verified the superiority of our model. The experiment took the TT100K dataset produced by Tsinghua University as the base dataset, followed by generalization tests on the CCTSDB 2021 dataset produced by Changsha University of Science and Technology.

### Introduction to the dataset

1) TT100K is a large-scale dataset jointly developed by Tsinghua University and Tencent to facilitate research in traffic sign detection for autonomous driving and intelligent transportation systems. The dataset contains approximately 10,000 high-resolution road images (2048 × 2048 pixels) annotated for object detection task, covering diverse urban, highway, and rural scenarios across multiple Chinese cities under various weather conditions and lighting situations. The dataset was divided into training, validation, and test sets in the ratio of 7:2:1.

**Fig 7:**
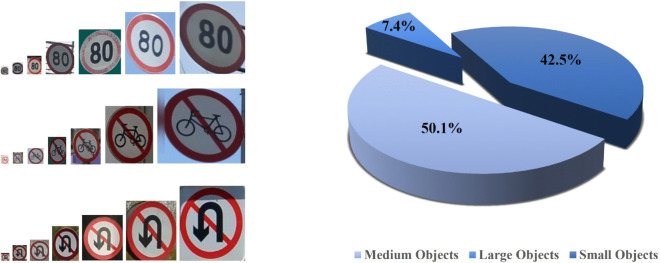
Data characteristics of the TT100K dataset.

As illustrated in Fig 7, traffic signs in TT100K present significantly greater scale variation compared to conventional datasets, with small objects (≤32 × 32 pixels) constituting 42.5 % of instances. This unique characteristic imposes stricter requirements on the model’s capability in detecting small-size and multi-scale targets. Moreover, a significant portion of the signs in TT100K are occluded, blurred, or severely affected by lighting conditions, which demands greater robustness from the model in complex scenarios.

2) CCTSD2021 was constructed by Changsha University of Science and Technology based on the CCTSD2017 traffic sign dataset by adding over 4,000 real traffic scene images. CCTSD2021 replaced many of the original easily detectable images with difficult samples to adapt to the complex and changeable detection environment. The dataset contains 17,856 images, among which there are 16,356 in the training set and 1,500 in the test set. The marked information is divided into three categories: prohibition, warning and sign. In addition, the labels were also classified according to the size of the target photo (XL, L, M, S, XS) and the weather conditions (cloudy, foggy, night, rainy, snow, sunny).

### Experimental environment

The proposed model was trained on an Ubuntu 20.04 system powered by an NVIDIA RTX 3090 GPU (24GB), leveraging PyTorch 2.2.2 with CUDA 12.1 acceleration. All input images were resized to 640×640 resolution and processed with a batch size of 32. We adopted an initial learning rate of 0.01, along with a momentum of 0.937 and weight decay of 0.0005. Training proceeded for 1,500 epochs, with early stopping triggered if validation performance did not improve within 100 epochs. All data underwent mosaic data augmentation. The complete set of hyperparameters is detailed in Table 1.

**Table 1:** Parameter specifications.

Batch	Imgsz	epochs	lr0	lrf	momentum	decay	patience
32	640 × 640	1500	0.01	0.01	0.937	0.0005	100

### Evaluation indicators

We adopt six standard evaluation metrics in object detection: Precision, Recall, mAP@50, mAP@50:95, Parameters, and GFLOPs, whose formal definitions are provided below:

$$Precision=\frac{TP}{TP+FP}$$ (24)

$$Recall=\frac{TP}{TP+FN}$$ (25)

$$AP=\int_{0}^{1}p(r)dr$$ (26)

$$mAP=\frac{1}{N}\sum_{i=1}^{N}AP_{i}$$ (27)

In the above formulas, precision quantifies the proportion of correctly predicted positive instances among all predicted positives. Formally, True Positives (*TP*) denote positive samples correctly predicted as positive, while False Positives (*FP*) represent negative samples incorrectly predicted as positive; *Recall* measures the fraction of actual positive instances correctly predicted as positive. In its formulation, *TP* again refers to correctly predicted positives, whereas False Negatives (*FN*) indicate positive samples erroneously predicted as negative; Average Precision (*AP*) is derived from the Area Under the Precision-Recall Curve (AUC-PR). For varying classification thresholds, *Precision* and *Recall* are computed, and the integral of the resulting P-R curve defines the *AP*. In its formulation, *r* denotes a point on the horizontal axis (*Recall*), p(r) represents the precision at a specific recall level and $\int_{0}^{1}p(r)dr$ p(r)dr corresponds to the Area Under the Curve (AUC), defined as the region bounded by the P-R curve, the horizontal axis (*Recall*), and the vertical axis (precision); Mean Average Precision (*mAP*) evaluates the model’s overall detection quality across all classes by averaging per-class *AP* value, where N denotes the total number of classes, and *AP*_*i*_ is the *AP* for the *i* class.

### Performance on the TT100K dataset

**Comparative Experiments on TT100K dataset**. To validate the effectiveness of our proposed improvements, we conducted comprehensive comparisons with state-of-the-art one-stage object detectors, including: YOLOv8n [9], YOLOv9t [35], YOLOv10n [36], YOLOv11n, YOLOv12n [37] and Hyper-YOLO-t [38]. Furthermore, we evaluated three state-of-the-art YOLO-based traffic sign detection models: CSW-YOLO-n [11] (optimized for small-object detection), SCB-YOLO [39] and a lightweight variants - GRFS-YOLO [10]. As evidenced by the results in Table 2, our proposed model ESA-YOLO surpasses all compared state-of-the-art methods in label prediction performance.

**Table 2:** Comparison with other models on the TT100K dataset.

Method	Year	Precision	Recall	mAP50	mAP50-95	Params	Gflops	Size
YOLOv8n [9]	2023	0.837	0.723	0.816	0.631	3.0M	8.1	6.4MB
YOLOv9t [35]	2024	0.823	0.707	0.806	0.62	1.9M	7.6	4.8MB
YOLOv10n [36]	2024	0.841	0.722	0.819	0.635	2.7M	8.1	5.8MB
YOLOv11n	2024	0.829	0.743	0.827	0.635	2.6M	6.4	5.6MB
Hyper-YOLO-t [38]	2024	0.832	0.716	0.809	0.623	3.6M	9.5	7.5MB
YOLOv12n [37]	2025	0.845	0.733	0.826	0.641	2.5M	6.4	5.6MB
SCB-YOLO [39]	2024	0.809	0.725	0.801	0.605	6.5M	16.4	13.4MB
GRFS-YOLO [10]	2024	-	**0.95**	0.712	0.548	1.7M	10.9	-
CSW-YOLO-n [11]	2025	0.821	0.742	0.832	0.639	2.5M	11.4	5.5MB
ESA-YOLO(Ours)	-	**0.865**	0.786	**0.865**	**0.674**	2.0M	6.7	4.9MB

Specifically, when compared with the latest YOLO detector, YOLOv12n, our model achieves superior performance with relative improvements of +5.3% in recall, +3.9% in mAP@50, and +3.3% in mAP@50:95. Furthermore, while maintaining comparable computational costs to the most lightweight counterparts (YOLOv11n and YOLOv12n), our model reduces parameter count and model size by approximately 20% and 13% respectively. Compared to the lightweight GRFS-YOLO, our proposed method not only achieves significantly better detection accuracy, with a +15.3% mAP@50 improvement, but also reduces computational cost by approximate 40%. Compared with CSW-YOLO-n, our model demonstrates comprehensive superiority - achieving 4.4% higher recall and 3.3% better mAP@50 while requiring only 80% parameters and 58% computational costs of CSW-YOLO-n.

Moreover, to validate the superior detection performance of our proposed DMFPN on small and multi-scale objects, we conducted comparative experiments with other advanced feature pyramid networks. We adopt YOLOv11 as the baseline model and systematically compare seven neck architectures: PANet-P345 [15], PANet-P2345 [15], BiFPN [16], RepGFPN [20], AFPN [19], HSFPN [22], MAFPN [21], and our proposed DMFPN for feature fusion. The experimental results are presented in Table 3.

**Table 3:** Comparison with FPN on the TT100K dataset.

FPN	Year	Precision	Recall	mAP50	mAP50-95	Params	Gflops	Size
PANet-P345 [15]	2018	0.829	0.743	0.827	0.635	2.6M	6.4	5.6MB
PANet-P2345 [15]	2018	0.829	0.754	0.84	0.655	2.7M	10.6	6.0MB
BiFPN [16]	2020	0.849	0.73	0.829	0.647	1.9M	6.3	4.3MB
RepGFPN [20]	2022	0.843	0.734	0.819	0.636	3.7M	8.2	8.0MB
AFPN [19]	2023	0.845	0.723	0.821	0.634	2.7M	8.9	5.8MB
HSFPN [22]	2024	0.826	0.725	0.813	0.632	1.9M	5.9	4.4MB
MAFPN [21]	2024	0.861	0.725	0.827	0.644	2.7M	7.1	5.9MB
DMFPN(Ours)	-	**0.866**	**0.76**	**0.849**	**0.661**	2.1M	6.7	4.7MB

Our experiment reveals that the PANet neck structure, a consistent component in the YOLO series, demonstrates superior generalization capability for traffic sign detection compared to newer FPN variants like AFPN, HSFPN and RepGFPN. Augmenting PANet with an additional small-object detection layer yields significant accuracy improvements (+1.3% mAP@50 and +2.0% mAP@50-95), validating the importance of small-object detection in traffic sign recognition. However, this approach incurs substantial computational overhead. BiFPN achieves better performance (+0.2% mAP@50 and +1.2% mAP@50-95) with reduced complexity, confirming the effectiveness of multi-path fusion and dynamic weighting. While MAFPN’s intensive fusion strategy improves mAP@50-95 by 0.9%, its overall performance trails BiFPN, suggesting potential channel redundancy across feature scales. Our proposed DMFPN outperforms all compared FPNs, delivering 2.2% higher mAP@50 than PANet-P345 and surpassing even the small-object-enhanced PANet-P2345 by 0.9% mAP@50 and 0.6% mAP@50-95, while requiring only 60% of its computational cost - demonstrating exceptional multi-scale and small-object detection efficiency.

**Ablation experiment on TT100K dataset**. In this section, we conduct ablation studies on the TT100K dataset using YOLOv11 as the baseline to evaluate the impact of each proposed improvement. As shown in Table 4, all modifications demonstrate consistent performance gains.

**Table 4:** Ablation experiment result on TT100K dataset.

Base	DMFPN	CAGB	ASPH	Precision	Recall	mAP50	mAP50-95	Params	Gflops	Size
✓				0.829	0.743	0.827	0.635	2.6M	6.4	5.6MB
✓	✓			0.866	0.76	0.849	0.661	2.1M	6.7	4.7MB
✓		✓		0.832	0.753	0.837	0.637	2.4M	6.2	5.5MB
✓			✓	0.839	0.743	0.833	0.646	2.6M	6.5	5.7MB
✓		✓	✓	0.843	0.753	0.832	0.651	2.4M	6.4	5.6MB
✓	✓	✓		**0.876**	0.747	0.853	0.668	2.0M	6.6	4.8MB
✓	✓		✓	0.855	0.775	0.857	0.665	2.1M	6.8	4.8MB
✓	✓	✓	✓	0.865	**0.786**	**0.865**	**0.674**	2.0M	6.7	4.9MB

Specifically, replacing the original neck with DMFPN yields a notable 2.2% mAP@50 and 2.6% mAP@50-95 improvement while reducing parameters by 0.5MB, indicating its superior multi-scale feature fusion capability for small object detection. Substituting the C3K2 feature extractor with our CAGB module improves mAP@50 by 1.0% while simultaneously reducing parameters and computations by 0.2M and 0.2 GFLOPs respectively, which indicates that the module we proposed has more excellent feature extraction capabilities and is more efficient. The ASPH brings 0.6% mAP@50 and 1.1% mAP@50-95 gain with negligible computational overhead. Subsequently, we conducted combined experiments on various improvements. First, we combined CAGB and ASPH, and the results showed that mAP@50-95 of the combined model increase 1.4% when CAGB was added alone. The combination of DMFPN with CAGB or ASPH further improved mAP@50 by 0.4% and 0.8%, respectively, compared with adding DMFPN alone. The final model incorporating all three innovations achieves optimal performance - 3.8% higher mAP@50 and 3.9% better mAP@50-95 than baseline, with 6M parameters and 0.7MB model size reduction.

**Fig 8:**
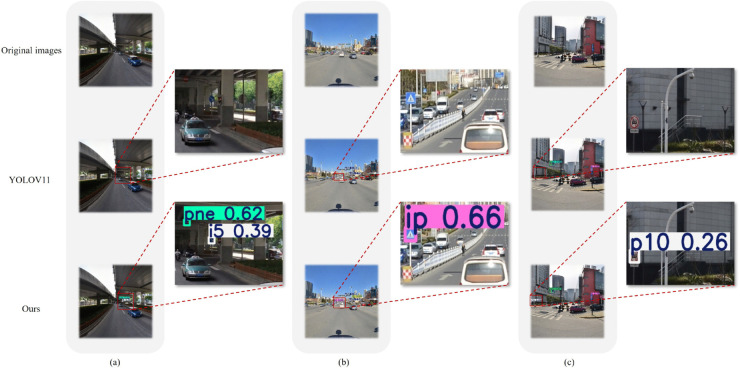
Visualization of the detection results of the TT100K dataset.

**Visualization result of detection on TT100K**. We visualize the detection results on TT100K as shown in Fig 8. As demonstrated in column (a) of the visualization results, our proposed model successfully detects two small traffic signs - ’No Entry’ and ’Keep Right’ under low-light conditions, which were missed by YOLOv11. Column (b) shows our model’s additional detection of a small ’Pedestrian Crossing’ sign in complex backgrounds. In particular, in column (c), our method accurately identifies the minimally visible sign ’No Motor Vehicles’ in the image periphery and demonstrates significantly higher detection confidence for significantly deformed ’pedestrian crossing’ signs appearing in the image regions of the right side, showing the robustness of our models to geometric distortion. These visual comparisons substantiate that our model exhibits enhanced capability for small-object detection and superior robustness in challenging scenarios.

**Fig 9:**
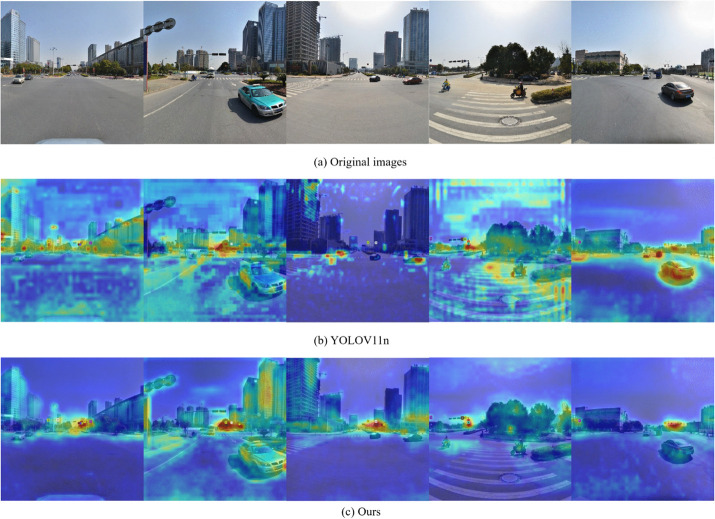
Comparison of heatmaps of YOLOv11 and ESA-YOLO.

The comparison of the heat map is shown in Fig 9. Heat maps are typically used in the field of computer vision to represent the importance or attention of a specific area. Usually, the highlighted parts are the positions that the model considers important. It can be seen that several heat maps of YOLOv11 show an irregular distribution of bright colors throughout the entire map, which indicates that its focus is chaotic and is severely disturbed by the background. For instance, for the last image, YOLOv11 focused a great deal of attention on the car in the background, which had little relevance to our detection task. On the contrary, the focus of ESA-YOLO can be well focused on the area where the traffic signs are located and is relatively less affected by the environment. Therefore, it is more likely to detect more targets to be detected. For example, for the second and third images, ESA-YOLO not only focuses on the area more precisely, And it successfully detected the traffic signs that YOLOv11 failed to detect.

### Performance on the CCTSDB2021 dataset

**Comparative Experiments on CCTSDB2021 dataset**. To validate the model’s generalization capability, we conduct cross-dataset evaluation on another public traffic sign detection dataset CCTSDB201. As shown in Table 5, our model achieves state-of-the-art performance, attaining the highest mAP@50 (81.7%) and mAP@50-95 (53.4%) among all compared methods. Compared to the baseline YOLOv11, our approach demonstrates significant improvements of +2.3% and +1.8% in mAP@50 and mAP@50-95 respectively, while maintaining competitive recall (73.2%), second only to Hyper-YOLO. Notably, YOLOv10 emerges as the second-best performer, likely benefiting from its innovative NMS-free design that effectively suppresses false positives. For traffic sign detection detectors, our model outperforms SCB-YOLO by +7.3% (mAP@50) and +8.2% (mAP@50-95), CSW-YOLO-n by +2.3% (mAP@50) and +1.8% (mAP@50-95) and GRFS-YOLO by +1.4% (mAP@50) and +1.6% (mAP@50-95). In terms of efficiency, our model achieves remarkable compactness with only 2.1M parameters - the smallest among comparable models except for the ultra-lightweight YOLOv9t (2.0M) and GRFS-YOLO (1.65M) and it requires only 6.6 GFLOPs, representing just 86% and 65% of the computational costs of YOLOv9t and GRFS-YOLO respectively, while delivering superior accuracy.

**Table 5:** Comparison with other models on the CCTSDB dataset.

Method	Year	Precision	Recall	mAP50	mAP50-95	Params	Gflops	Size
YOLOv8n [9]	2023	0.878	0.721	0.792	0.501	3.0M	8.1	6.4MB
YOLOv9t [35]	2024	0.86	0.721	0.794	0.505	2.0M	7.6	4.8MB
YOLOv10n [36]	2024	0.883	0.716	0.807	0.53	2.7M	8.1	5.8MB
YOLOv11n	2024	0.884	0.725	0.794	0.516	2.6M	6.3	5.5MB
Hyper-YOLO-t [38]	2024	0.849	**0.737**	0.801	0.514	3.6M	9.5	7.5MB
YOLOv12n [37]	2025	0.856	0.723	0.783	0.502	2.6M	6.3	5.5MB
SCB-YOLO [39]	2024	0.862	0.65	0.744	0.452	6.5M	16.4	13.3MB
GRFS-YOLO [10]	2024	-	0.724	0.803	0.518	1.65M	9.8	-
CSW-YOLO-n [11]	2025	0.875	0.725	0.794	0.516	2.4M	10.1	5.3MB
ESA-YOLO(Ours)	-	**0.903**	0.732	**0.817**	**0.534**	2.1M	6.6	4.9MB

To validate our model’s robustness in complex driving scenarios, we conducted experiments on the CCTSDB2021 dataset, which categorizes images by environmental conditions. The results in Table 6 demonstrate our model’s superior adaptability across diverse challenging scenarios.

**Table 6:** Comparison with other models under different weather conditions.

Method	Night		Rain		Foggy		Cloud		Sunny		Snow	
	Recall	map50	Recall	map50	Recall	map50	Recall	map50	Recall	map50	Recall	map50
YOLOV8n [9]	**0.711**	0.772	0.419	0.31	**0.697**	0.744	0.826	0.908	0.865	0.926	0.609	0.725
YOLOV9t [35]	0.69	0.753	0.409	0.472	0.472	0.705	0.836	0.905	0.846	0.923	0.722	0.78
YOLOV10n [36]	0.703	0.765	0.41	0.505	0.54	0.708	0.852	0.911	0.878	0.937	0.729	0.812
YOLOV11n	0.689	0.756	0.418	0.456	0.613	0.646	0.825	0.904	0.862	0.937	0.747	0.829
Hyper-YOLO-t [38]	0.695	0.76	0.436	0.356	0.613	0.714	**0.855**	0.913	0.836	0.919	0.714	0.828
YOLOV12n [37]	0.668	0.722	0.424	0.388	0.559	0.647	0.842	0.898	0.878	0.928	0.727	0.772
SCB-YOLO [39]	0.586	0.662	0.235	0.266	0.633	0.72	0.784	0.861	0.82	0.912	0.719	0.797
CSW-YOLO-n [11]	0.685	0.772	0.57	0.51	0.691	**0.795**	0.828	0.889	0.864	0.934	0.696	0.769
ESA-YOLO(Ours)	0.693	**0.781**	**0.595**	**0.559**	0.678	0.735	0.822	**0.914**	**0.879**	**0.942**	**0.828**	**0.897**

Specifically, in night scenes, our model achieves the highest mAP@50 (78.1%), outperforming baseline YOLOv11 by +2.5% while YOLOv8n excels in recall (0.711%) and ranks second in mAP@50 (0.772%). In rain scenes, our model delivers optimal recall (0.595) and mAP@50 (0.559), surpassing YOLOv11 by +17.7% (recall) and +10.3% (mAP@50). CSW-YOLO performs second-best, significantly exceeding other mainstream models. In foggy scenes, YOLOv8n achieves the highest recall (0.697%), while CSW-YOLO-n leads in mAP@50 (0.795%). Our model ranks third, improving upon YOLOv11 by +6.5% (recall) and +8.9% (mAP@50). In cloud scenes, our model attains the highest mAP@50 (0.914%), +1% gain over YOLOv11. Hyper-YOLO-t shows the best recall (0.855%), closely followed by YOLOv10 (0.852%). In sunny scenes, our model dominates with the highest recall (0.879%) and mAP@50 (0.942%), improving baseline performance by +1.7% (recall) and +0.5% (mAP@50). In snow scenes, our model significantly outperforms competitors with recall (0.828%) and mAP@50 (0.897%)—+8.1% (recall) and +6.8% (mAP@50) over YOLOv11, which is the second-best model. To investigate the underlying causes of this phenomenon, we conducted a analysis of the CCTSDB dataset in snowy weather conditions. Our evaluation revealed a notably high proportion of small-scale traffic signs within this subset. This observation suggests that the improved small-target detection capability of our model contributes to its superior performance in such challenging scenarios. Our model exhibits superior robustness under night, rain, foggy, cloudy, sunny, and snowy conditions, demonstrating its suitability for real-world autonomous driving environments.

To further validate our model’s small object detection capability, we conducted comparative experiments using the "XS" and "S" subsets from the CCTSDB dataset, which demonstrating "Very Small" and "Small" respectively. As shown in Table 7, our model achieves the highest Precision (88.2%) and mAP@50 (78.2%) for very small objects, outperforming the baseline YOLOv11n by 8.2% and 4.3%, respectively. CSW-YOLO-n, which also enhances the small object detection capability, achieves the highest Recall (71.9%), while YOLOv10n obtains the best mAP@50-95 (38.5%). For small objects, our model demonstrates superior performance with the highest mAP@50 (90.8%) and mAP@50-95 (58.5%), surpassing all other compared models by a significant margin—3.5% and 2.7% higher than YOLOv11n, respectively. Additionally, our model also leads in Precision (92.5%), with YOLOv10n (92.3%) and CSW-YOLO-n (92.2%) following closely. YOLOv12n, however, achieves the highest Recall (82.7%). Overall, for very small objects, YOLOv10n and CSW-YOLO-n also exhibit strong detection performance alongside our model, whereas for small objects, our approach demonstrates a dominant advantage. These results clearly indicate that the proposed improvements effectively enhance the model’s capability in detecting small-scale targets.

**Table 7:** Comparison with other models under different size.

Method	XS				S			
	Precision	Recall	map50	map50- 95	Precision	Recall	map50	map50-95
YOLOV8n [9]	0.814	0.617	0.682	0.291	0.909	0.779	0.86	0.534
YOLOV9t [35]	0.798	0.689	0.736	0.353	0.884	0.798	0.856	0.541
YOLOV10n [36]	0.807	0.707	0.77	**0.385**	0.923	0.769	0.874	0.572
YOLOV11n	0.8	0.679	0.739	0.34	0.917	0.806	0.873	0.558
Hyper-YOLO-t [38]	0.837	0.649	0.751	0.342	0.888	0.809	0.875	0.55
YOLOV12n [37]	0.735	0.65	0.689	0.323	0.898	**0.827**	0.85	0.524
SCB-YOLO [39]	0.81	0.508	0.612	0.244	0.875	0.724	0.808	0.468
CSW-YOLO-n [11]	0.833	**0.719**	0.777	0.373	0.922	0.772	0.853	0.554
ESA-YOLO(Ours)	**0.882**	0.703	**0.782**	0.359	**0.925**	0.774	**0.908**	**0.585**

**Fig 10:**
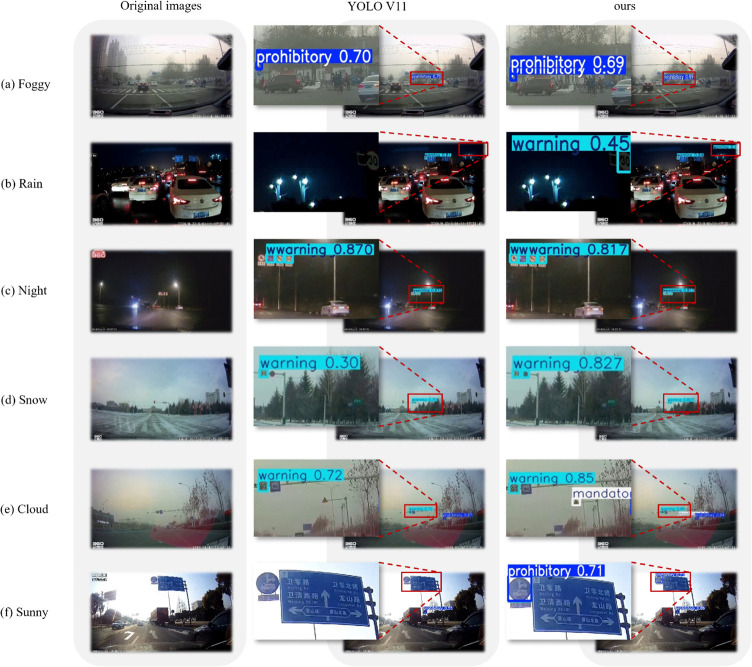
Visualization of the detection results of the CCTSDB dataset under different weather conditions.

**Visualization result of detection on CCTSDB**. In this section, we present a visual comparison to demonstrate the superior performance of our proposed model across diverse challenging scenarios (foggy, rain, night, snow, cloud and sunny). As illustrated in Fig 10, each comparison group consists of three columns: The first column displays the original scene, while the second and third columns show the detection results from the baseline YOLOv11n model and our proposed model, respectively. For the foggy image in row (a), our model successfully detects a "Prohibitory" sign missed by YOLOv11n. For the rainy image in row (b), our model identifies an additional "Warning" sign under low illumination, which YOLOv11n fails to detect. For the nighttime image in row (c), our model captures all densely arranged small-scale "Prohibitory" signs in low-light conditions, whereas YOLOv11n exhibits significant detection failures. For the snowy image in row (d), our model detects an extremely small "Prohibitory" sign overlooked by YOLOv11n, with substantially higher confidence scores. For the cloudy image in row (e), our model demonstrates superior capability in recognizing " Mandatory" signs with reduced contrast due to weather degradation. For the sunny image in row (f), while YOLOv11n detects a small "Prohibitory" sign, it misses a standard-sized counterpart under strong illumination – both are accurately identified by our model with notably higher confidence. These visual comparisons empirically validate that our model achieves significantly improved robustness in complex environments, demonstrating stronger applicability for real-world autonomous driving systems where environmental variability is critical.

## Conclusion

The paper presents an improved YOLOv11-based traffic sign detection algorithm that addresses key challenges in autonomous driving systems, including multi-scale object detection, small-target recognition, and robustness in complex environments. Through systematic architectural enhancements, the proposed model achieves superior performance while maintaining computational efficiency. The DMFPN significantly enhances multi-scale feature fusion by enabling comprehensive bidirectional interaction between high-level semantic and low-level spatial information. The CAGB effectively integrates local and global contextual information through computationally efficient token and channel mixers, enhancing small-object detection ability without excessive parameter overhead. The ASPH synergistically combines multi-scale feature extraction with attention mechanisms to enhance robustness in adverse conditions. Experimental evaluations on TT100K and CCTSDB2021 datasets demonstrate the model’s state-of-the-art performance and exceptional generalization across challenging conditions. In most complex scenarios (rain, night, snow and sunny), ESA-YOLO demonstrates stronger robustness. However, in certain specific cases—such as foggy environments, there is still a noticeable decrease in accuracy. Overall, this paper provides a practical solution for traffic sign detection in autonomous driving, balancing accuracy, efficiency, and robustness.

Although the proposed model achieves a favorable balance between efficiency and accuracy, it still exhibits certain limitations. ESA-YOLO exhibits suboptimal detection performance in individual complex scenarios, indicating that its robustness requires further enhancement. Moreover, due to limitations in the dataset, the model achieves reliable detection primarily for the 45 most common traffic sign categories. Therefore, in future work, I plan to collect images in specific complex scenarios—such as hazy conditions where the model currently underperforms—to augment the dataset. Additionally, I will explore applying image preprocessing techniques, such as image dehazing, to enhance image quality, thereby improving the model’s robustness in these challenging environments. Furthermore, I intend to source or collect a wider variety of traffic sign images, especially for categories that are underrepresented in existing public datasets, in order to further enrich the dataset and enable more accurate recognition across a broader range of classes.
